# Synthesis of Zeolitic Imidazolate Framework-8 Using Glycerol Carbonate

**DOI:** 10.1021/acssuschemeng.3c02876

**Published:** 2023-08-21

**Authors:** Masaki Itatani, Norbert Német, Nadia Valletti, Gábor Schuszter, Prisco Prete, Pierandrea Lo Nostro, Raffaele Cucciniello, Federico Rossi, István Lagzi

**Affiliations:** † Department of Physics, Institute of Physics, Budapest University of Technology and Economics, Műegyetem rkp. 3, H-1111 Budapest, Hungary; ‡ Department of Organic Chemistry and Technology, Budapest University of Technology and Economics, Műegyetem rkp. 3, H-1111 Budapest, Hungary; § Department of Physical Sciences, Earth and Environment, Univeristy of Siena, Piazzetta Enzo Tiezzi 1, 53100 Siena, Italy; ∥ Department of Physical Chemistry and Materials Science, University of Szeged, Rerrich Béla tér 1, H-6720 Szeged, Hungary; ⊥ Department of Chemistry and Biology, University of Salerno, viale Giovanni Paolo II 132, Fisciano, Salerno 84084, Italy; # Department of Chemistry “Ugo Schiff”, University of Firenze, via della Lastruccia 3, Sesto Fiorentino, Florence 50019, Italy; ¶ Centro Interdisciplinare Linceo Giovani, Accademia Nazionale dei Lincei, Via della Lungara, 10, 00165 Roma, Italy; ∇ ELKH-BME Condensed Matter Research Group, Budapest University of Technology and Economics, Műegyetem rkp. 3, H-1111 Budapest, Hungary

**Keywords:** metal−organic framework (MOF), zeolitic imidazolate framework (ZIF), recycle, solvents, porous materials

## Abstract

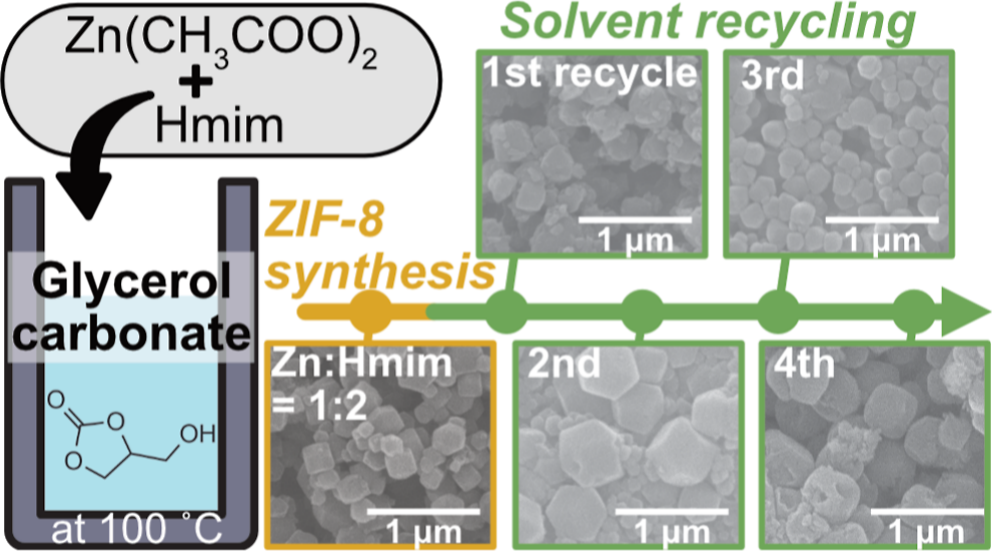

In this study, we show that glycerol carbonate (GlyC), a bio-based derivative of glycerol, can be used as a suitable green solvent for the synthesis of metal–organic frameworks (MOFs). In particular, a zinc-based zeolitic imidazolate framework-8 (ZIF-8) was synthesized by exploring several different experimental conditions (in terms of temperature, reaction time, and reactants’ concentrations) to find that the yield of the reaction and the quality of the products, measured in terms of crystallinity, surface area, and porosity, were in line with those obtained in the most commonly (non-green) used solvents. GlyC was also found to be reusable for several cycles, maintaining the same original quality as a solvent for the synthesis. Finally, some indicators for the assessment of the greenness of a process (E-factor and PMI) revealed a milder environmental impact of GlyC with respect to other solvents.

## Abstract 2

Glycerol carbonate can be used as a green solvent for synthesizing zeolitic imidazole framework-8, which can be also recycled for several cycles.

## Introduction

Zeolitic imidazolate framework-8 (ZIF-8) is one of the representative materials of metal–organic frameworks (MOFs),^1^ a class of materials composed of metal cations coordinating organic ligands. Due to their special structure, MOFs have a high specific surface area and large pore size, which render them a good candidate for gas storage, separation, chromatography, electronic applications, and drug delivery.^2−5^ In this respect, many efforts were made to control the ZIF-8 crystal morphology and crystal size distribution. To pursue this goal, the control of crystal size distribution was achieved using different strategies such as the adjustment of the reactant ratios, the incorporation of additives (e.g., trialkyl amine, cetyltrimethylammonium bromide, formate, etc.), the sources of Zn(II) ions, and the proper selection of the synthetic method (sono-crystallization, micromixer, ionothermal microwave-assisted synthesis, microwave irradiation).^6^ In contrast, the research on new green and bio-based solvents for MOF synthesis is still limited. Indeed, methods using amide-type solvents (e.g., dimethylformamide, DMF) are the most used for the synthesis of ZIF-8^7^ with scant examples based on more environment-friendly alternatives, such as methanol^8^ or water.^9^ When water is used as a solvent, the synthesis should be carried out in an alkaline environment usually by applying an excess of organic linkers.^10−13^ Rapid synthesis methods (<1 h) at room temperature are also presented in the literature, however, in this case, the size of the formed crystals is less than 100 nm.^14^ In fact, DMF is a toxic, fossil-based, polar, and aprotic solvent characterized by a high dielectric constant and a high boiling point, features that favor the progress of the synthetic process^15^ but may pose concerns on the environmental impacts.

To overcome this limitation and with the ambitious idea to substitute DMF with greener alternatives, in this work we report for the first time the preparation of crystalline ZIF-8 in glycerol carbonate (GlyC). GlyC (4-hydroxymethyl-2-oxo-1,3-dioxolane) has attracted increasing interest as one of the most investigated bio-based compounds with many interesting applications in different fields, such as in the synthesis of new chemicals,^16,17^ the manufacturing of polymers, building blocks for drug preparation, surfactants, cosmetics, and detergent industries. Moreover, the use of GlyC fully respects the 7th principle of Green Chemistry^17−22^ due to its preparation from glycerol through transcarbonation with dimethyl carbonate. Because of its physicochemical properties (Table S1 in Supporting Information), GlyC is also used as a biodegradable, low-volatile organic solvent with low toxicity having a high dielectric constant and dipole moment.^23^ Indeed, GlyC shows relevant and promising properties to overcome the limitations related to DMF for ZIF-8 preparation. In this work, to further assess the advantages concerning the use of GlyC for this purpose, solvent recyclability experiments were carried out, and both simple E-Factor (sEF) and process mass intensity (PMI) were calculated and discussed.

## Experiments

### Regents and Materials

Dimethyl carbonate (DMC), glycerol (>99%), sodium carbonate (Na_2_CO_3_), zinc acetate dihydrate (>99%, Zn(OAc)_2_·2H_2_O), 2-methylimidazole (99%, Hmim), sodium hydroxide (>98%, NaOH), dimethyl sulfoxide (DMSO), and DMF were purchased from Merck (Darmstadt, Germany).

### Synthesis and Characterization of GlyC

GlyC was prepared by glycerol transcarbonation with DMC in the presence of Na_2_CO_3_ as a catalyst.^24,25^ 900 g (10.0 mol) of DMC, 300.6 g of glycerol (3.34 mol), and 1.06 g of Na_2_CO_3_ (1.0 mol) were introduced into a 2 L three-necked flask equipped with a mechanical stirring, a steam condenser, and a temperature sensor. The mixture was stirred and refluxed (75 °C) for 2 h. Afterward, the catalyst was filtered off and the excess of dimethyl carbonate and the produced methanol were distilled under reduced pressure. GlyC was obtained as a colorless viscous liquid (394 g) with a purity of 96% [nuclear magnetic resonance (NMR) analysis]. The NMR spectra were collected with a Bruker AVANCE-400 (100 ^13^C) spectrometer using deuterated DMSO as the solvent (for details see the Supporting Information).

### Synthesis and Characterization of ZIF-8

A given amount of Zn(OAc)_2_·2H_2_O and Hmim were dissolved separately in the synthesized pure GlyC or GlyC containing sodium hydroxide (0.01 M) by sonicating and heating at 60–70 °C, respectively. The reason for the addition of sodium hydroxide is that it favors the formation of ZIF-8 crystals not only in water but also in less polar solvents.^26,27^ Then, these mixtures were allowed to stand at room temperature to cool down, and each solution was degassed using N_2_ stream. The two solutions were then mixed. The reaction was carried out in glass vials at three different temperatures, namely room temperature (∼20 °C), 60, and 100 °C, using a heating block. Vials were covered with septum caps. The precipitate was allowed to settle for different periods of time (1, 2, and 7 days) and the generated particles were collected by centrifugation at 7000 rpm, rinsed one time with water and three times with DMF to remove unreacted chemical species. In the recycle experiments, the solvent was collected after the first centrifugation, filtered through an agarose membrane (0.45 μm pore size) to remove large particles and aggregates and finally used for a new synthesis in an iterative procedure.

To characterize the solid precipitate samples, the microstructure was investigated by scanning electron microscopy (SEM) and the crystalline phase assignment relied on powder X-ray diffraction (PXRD) measurements. The nitrogen adsorption data evaluated via Brunauer–Emmett–Teller (BET) and Barrett–Joyner–Halenda (BJH) methods provided us with the specific surface area and pore size distribution, respectively. A Hitachi S-4700 instrument was used at a 20 kV acceleration voltage and 10 μA current to take SEM micrographs. Prior to that, the samples were spread on a carbon tape, and gold sputtering (180 s, 18 mA) was applied to increase their conductance. PXRD diffractograms were registered by a Rigaku MiniFlex II desktop X-ray diffractometer at room temperature. The solid samples were spread on a silicon crystal cut holder to minimize the background noise. The measurements were conducted with a 30 kV accelerating voltage and 15 mA current. Scanning speed was set to 4°/min within 2Θ = 5–50° range applying 0.02° step size. As a radiation source, we used a standard Cu Kα (λ = 0.1542 nm) beam. Before the nitrogen adsorption measurements, samples were degassed for 2 h at 180 °C (previous studies showed that higher temperature degraded the samples’ microstructure). Nitrogen adsorption data were collected with a Nova 3000 (Quantachrome, USA) instrument. The range of 0.05–0.35 relative pressure was used to determine the specific surface area, and the desorption band of the isotherm was applied for the BJH method.

### Calculation of sEF, PMI, and Yield

Concerning the preparation of crystalline ZIF-8 and to enlarge the discussion about the reaction greenness, sEF and PMI calculations were carried out. Data obtained in the presence of GlyC as a solvent were counterposed to those reported in the literature for DMF^28^ and water.^10^ PMI values were also recalculated after the recovery of all the chemicals (PMIr).^29^
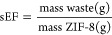
1
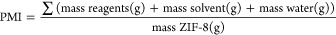
2
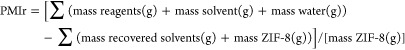
3

Also, a mass yield of synthesized ZIF-8 particles in GlyC was calculated by the following equation
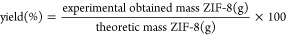
4where the “theoretic mass of ZIF-8” is the theoretic yield calculated by a given amount of limited reagent (namely, zinc ions in this study) and the empirical formula of ZIF-8, namely Zn(Hmim)_2_.

## Results and Discussion

**Figure 1:**
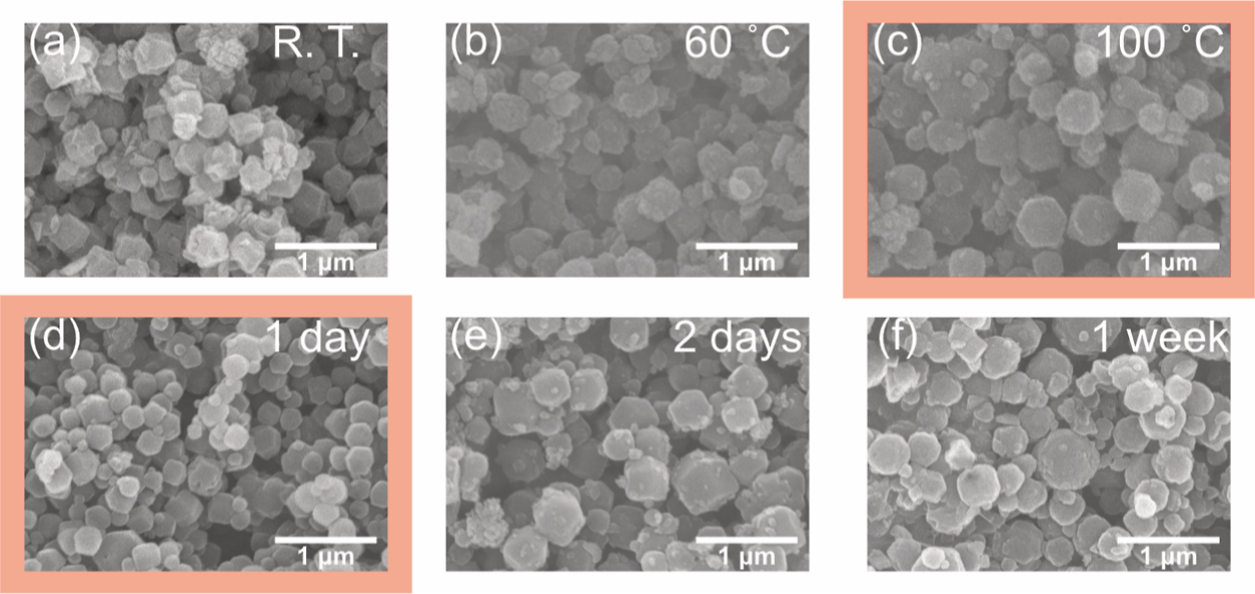
SEM micrographs of synthesized ZIF-8 after 2 days of reaction using 10 mM of NaOH at various temperatures: (a) room temperature (∼20 °C), (b) 60, and (c) 100 °C. Generated ZIF-8 at 100 °C using 10 mM of NaOH for various synthesis times: (d) 1 day, (e) 2 days, and (f) 1 week. All reactions were carried out using [Zn(OAc)_2_] = 10 mM and [Hmim] = 40 mM. Panels (c,d) are framed in orange to highlight the optimal conditions in terms of temperature and reaction time.

First, some preliminary experiments were carried out to obtain the optimal experimental conditions for the synthesis. We investigated the effect of temperature and of the total reaction time using fixed initial concentrations for the reactants: 10 mM zinc salt and four-fold excess of Hmim ([Zn(OAc)_2_] = 10 mM and [Hmim] = 40 mM).^30−33^ SEM micrographs in Figure 1 show that crystals having the typical geometry of ZIF-8 form in all experimental conditions when GlyC is used as the reaction medium. In particular, Figure 1a–c shows that the high temperature favors the formation of bigger and more crystalline particles. Concerning the reaction time, we found that 1 day was the optimal lapse, as illustrated in Figure 1d–f. This finding is in good accordance with the most widely applied synthesis time of ZIFs in hydro- and solvothermal methods.^34^ An increased reaction time would leads to the formation of bigger particles on average; however, after 2 days, the bigger crystals were covered by smaller particles (Figure 1e). After 1 week of reaction, the edges of the crystals became less sharp due to the degradation of the sample.^35^ Based on these experiments, the optimal experimental conditions for the generation of ZIF-8 in GlyC were found to be the same as those obtained in other solvents, namely high temperature and synthesis time of 1 day.^1,36^

**Figure 2:**
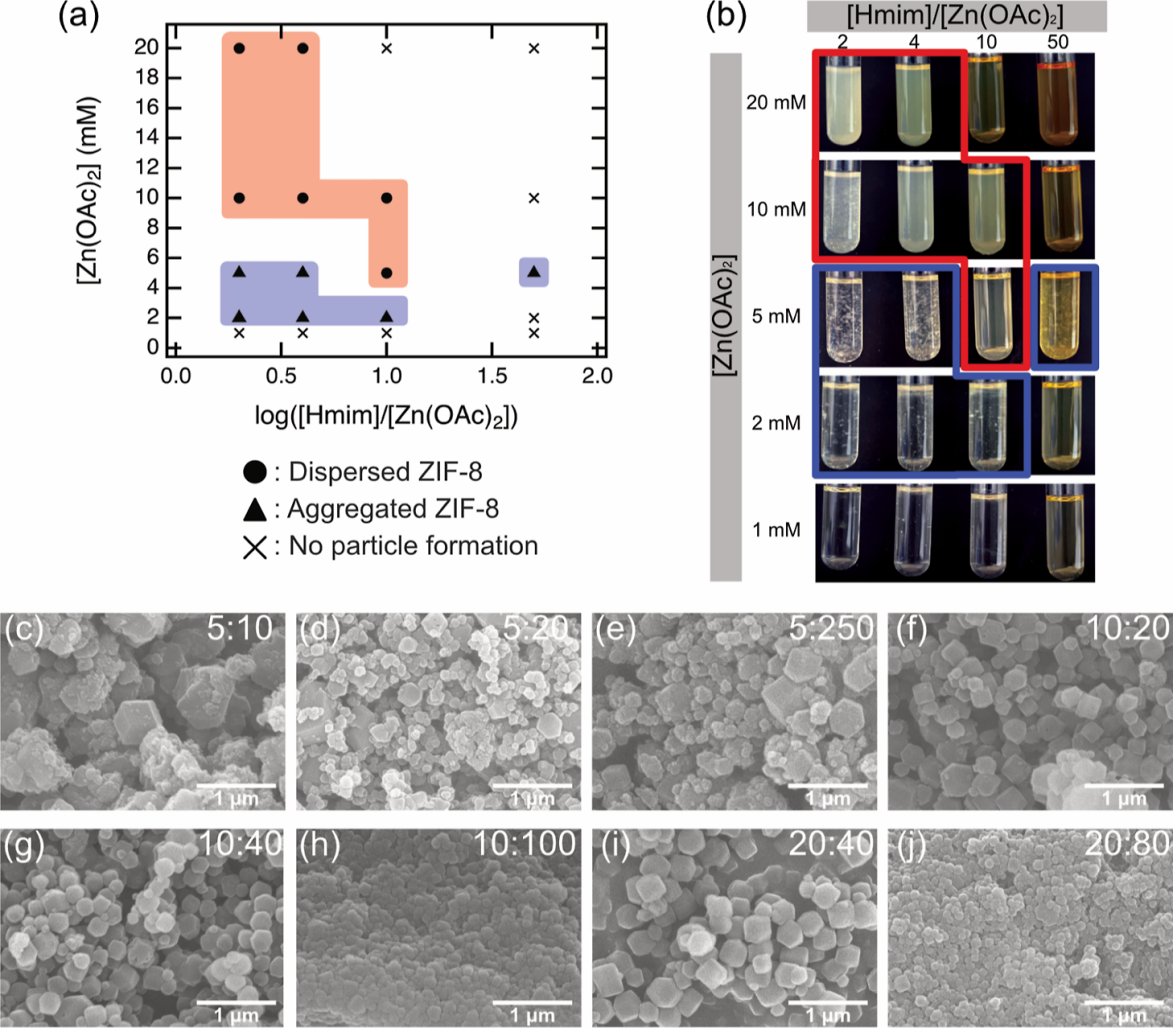
(a) Effect of the initial concentration of the reactants on the reaction and (b) photographs of the reaction mixture after 1 day at 100 °C. (c) SEM micrographs of ZIF-8 particles at Θ = 100 °C, *t* = 1 day, and [NaOH] = 0.01 M using [Zn(OAc)_2_]/[Hmim] = (c) 5:10 mM, (d) 5:20 mM, (e) 5:250 mM, (f) 10:20 mM, (g) 10:40 mM, (h) 10:100 mM, (i) 20:40 mM, and (j) 20:80 mM, respectively.

The next step was to explore whether the concentration of the reactants affects the size and the morphology of the ZIF-8 crystals, keeping the same experimental conditions (Θ = 100 °C, *t* = 1 day). Figure 2a summarizes the visual appearance of the colloidal suspension and the morphology of the crystals characterized by SEM measurements. At a low concentration of zinc ions (1 mM), no crystallization was observed. Based on the SEM measurements, the optimal crystallinity was obtained with the ratios [Zn(OAc)_2_]/[Hmim] = 10:20 mM, 10:40 mM, and 20:40 mM (Figure 2f,g,i). This is an interesting finding because, in most of the studies, the ratio of Hmim to Zn to generate ZIF-8 is greater than the stoichiometric ratio, which is 2:1. So far, the synthesis of ZIF-8 with a stoichiometric ratio of reactants was achieved using either a great amount of base^37^ or a jet-mixer reactor.^38^ In contrast, GlyC allowed the synthesis of ZIF-8 at the stoichiometric ratio, in mild reaction conditions, and using the simple solvothermal setup. Also, at the ratio of [Zn(OAc)_2_]/[Hmim] = 10:20 mM, the mass yield of the product was 57.1%, which is comparable to or even higher than that obtained using the common solvothermal setup with DMF and methanol as solvents.^28,36^ Further investigations on the crystalline phase assignment, specific surface area, pore volume, and recyclability measurements were conducted with a ratio of [Zn(OAc)_2_]/[Hmim] = 10:20 mM, i.e., at the lowest concentration of Hmim.

After the synthesis at a high concentration of Hmim (>100 mM), the solution turned to a yellowish color, as reported in Figure 2b. This was likely due to reactions of Hmim with the base and of GlyC with Hmim. In detail, the reaction of Hmim with NaOH leads to the formation of sodium 2-methylimidazolate which is the active organic ligand for ZIF-8 synthesis. This molecule shows significant absorption in the UV–vis range, depending on the solvent. Moreover, preliminary experiments carried out using sole Hmim and GlyC reveal the formation of side products which will be characterized in future work (more details in the Supporting Information). In passing, we recall that GlyC undergoes a yellowing degradation in the presence of bases.^39^ However, in the optimal concentration range for the formation of ZIF-8 crystals, the effect of the side reactions was negligible due to the relatively low concentrations of both cations and linkers.

**Figure 3:**
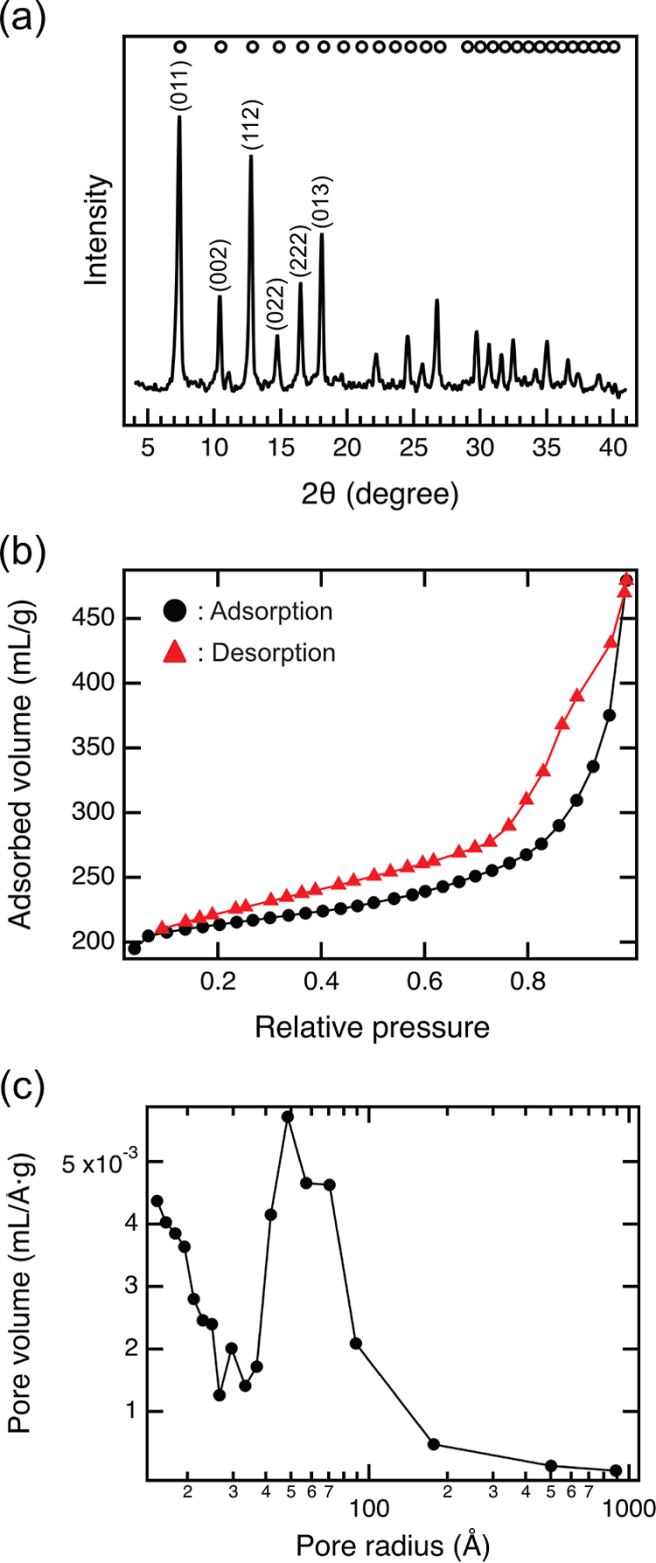
(a) PXRD pattern, (b) BET isotherm, and (c) BJH pore size distribution of ZIF-8 synthesized at 100 °C for 1 day with [Zn(OAc)_2_] = 10 mM, [Hmim] = 20 mM, and [NaOH] = 10 mM. The open circles above the peaks in panel (a) indicate the reported diffractions of ZIF-8.^37^

To characterize the solid products, we performed PXRD and nitrogen adsorption measurements (Figure 3). The recorded PXRD pattern showed excellent crystallinity and a good match with the data reported in the literature (Figure 3a).^37^ The average specific surface area of the ZIF-8 crystals was found to be 660 m^2^ g^–1^ which is roughly 50% less than the usual value of the samples synthesized in other organic solvents, e.g., DMF.^1,36,40^ The isotherm resembles type IV isotherms without the plateau near unity relative pressure. Type IV isotherms are characteristic of mesoporous materials, where the typical pore diameter, *d*, is between 20 and 500 Å. The absence of the plateau and thus the unlimited growth of the absorbed volume at higher relative pressures is an indicator of the presence of macropores (*d* > 500 Å). The same conclusion can be drawn from Figure 3c, i.e., the most characteristic pore size is ca. 50 Å, which is representative of mesoporous ZIFs.^12^ The distribution is relatively wide, and the population of pores of 40–100 Å diameter is equally important. Macropores ranging from 500 to 1000 Å can be found, but their abundance is low. Due to the features of the measurement, we do not consider the pores below 20 Å. The type H3 hysteresis is characteristic of pores surrounded by plates. Since the *dia* (diamond-like) polymorph of ZIF-8 is plate-like and less porous than the sodalite-like (SOD) polymorph, the hysteresis loop together with the relatively low specific surface area suggests that the *dia* polymorph is also present in the sample. The total pore volume is 0.58 cm^3^ g^–1^ (Figure 3b).

**Figure 4:**
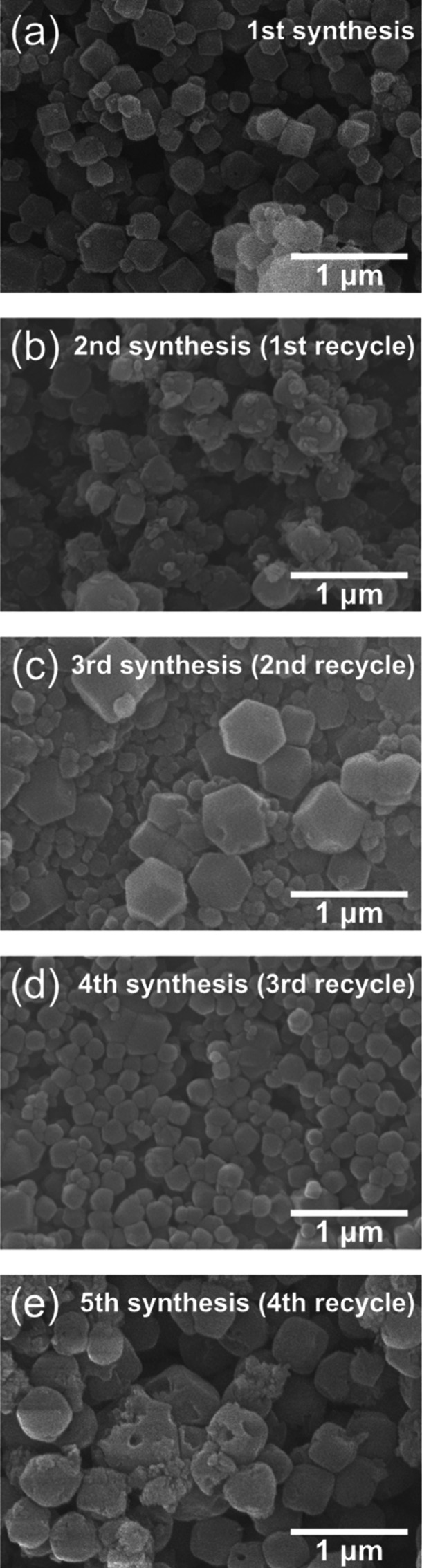
SEM micrographs of ZIF-8 synthesized in the first synthesis (a) and each recycle step: (b) first, (c) second, (d) third, and (e) fourth recycles. ZIF-8 was synthesized for every recycling experiment at 100 °C for 1 day with [Zn(OAc)_2_] = 10 mM, [Hmim] = 20 mM, and [NaOH] = 10 mM.

According to the circular economy approach, the recyclability of the solvent is one of the crucial issues that must be considered.^13,27,41,42^ We performed the sequential synthesis of ZIF-8 by reusing the same sample of GlyC as a solvent for several cycles. SEM measurements (Figure 4) and yield calculations based on eq 4 revealed that the formation of crystals with different yields varied from the first to fourth cycle: 52.8% (first), 58.8% (second), 62.8% (third), and 74.1% (fourth cycle), respectively. Increasing yield could be due to remaining and accumulating unreacted species (Zn^2+^ and Hmim) in the system after each cycle.

PMI has been proposed as a key mass-based metric.^43^ In the last years, several studies concerning the adoption of PMI have been published to analyze the process’s efficiency in terms of mass.^44^ We used both sEF and PMI not only to critically address the impacts of GlyC utilization and recycling but also to compare the ZIF-8 preparation in the presence of different solvents (GlyC, water, and DMF). However, it was recognized that these metrics did not address concerns regarding the toxicity and safety of the feedstock materials or wastes.^45^ The preparation of ZIF-8 in GlyC is performed with a sEF value of 1.2 after five consecutive reaction cycles. Indeed, the possibility to fully recycle the reaction mixture during five consecutive cycles gives an sEF, for each of these cycles, equal to 0 in agreement with the requirements of the circular approach.^41^ Results obtained in DMF and water gave an sEF of 9.4 and 11.2, respectively. These outcomes can be related to the lower ZIF-8 yields in DMF (30.5% w/w) using an Hmim/Zn^2+^ mole ratio of 8^28^ and to a high excess of Hmim (Hmim/Zn^2+^ mole ratio of 40) in water (ZIF-8 yield = 97.5% w/w).^10^ The sEF did not address the impact of water and solvent as, instead, PMI does. Therefore, PMI values are higher than sEFs. PMI values, calculated on the first reaction cycle to compare the three different scenarios, are 1122 for GlyC, 67 for DMF, and 281 for water. Considering the GlyC recyclability the PMI calculated after five reaction cycles is 206. Furthermore, considering the GlyC recyclability, the PMI reaches the value of 2.8.^29^ The obtained results clearly indicated the excellent impact of GlyC on the ZIF-8 synthesis by reducing the production of waste and through its recycling along four consecutive syntheses.

## Conclusions

In conclusion, we showed how an effective synthesis of technologically relevant materials, such as MOFs, can be successfully obtained in the bio-based solvent GlyC. Table 1 summarizes the characteristics of the ZIF-8 samples by using various methods of synthesis.^1,36,46−49^ The largest crystals (up to 200 μm) can be obtained in a solvothermal method using DMF. However, the microfluidic method generates particles with the largest specific surface area. The pore volume in all techniques spans between 0.4 and 0.7 cm^3^ g^–1^. In terms of yield and quality of the products, GlyC proved to be in line with the performances of other solvents, such as DMF and water, but having a milder environmental impact, as calculated by the sEF and the PMI. From a circularity point of view, the use of a derivative of glycerol for industrial applications can boost the use of bio-based chemicals and help the transition toward a more sustainable society.

**Table 1:** Comparison of the Sample Characteristics Obtained in Various Methods and Solvents for the Synthesis of ZIF-8^a^

synthesis method	particle size (μm)	*S*_BET_ (m^2^ g^–^^1^)	*V*_pore_ (cm^3^ g^–^^1^)	refs
solvothermal (DMF)	150–200	1370	0.51	(1,36)
solvothermal (MeOH)	3–5	1549	0.59	(36)
hydrothermal (H_2_O added TEA)	0.1–1	1340		(46)
microwave-assisted (DMF)	5–10	1250	0.53	(36)
microwave-assisted (MeOH)	0.33	61		(1,47)
sonochemical (DMF added TEA)	0.3–0.5	1249	0.71	(36)
mechanochemical	3–15	1256	0.64	(36)
microfluidic synthesis (DMF)	5–15	1435	0.42	(36)
microfluidic synthesis (H_2_O)	0.3–0.9	1730		(1,47)
dry-gel conversion (H_2_O)	0.3–0.4	1306	0.52	(36)
electrochemical	<1.0	1500	0.60	(1,47)
steam-assisted conversion (H_2_O)		1470		(1,49)
solvothermal (GlyC)	0.2–0.8	660	0.58	

a *S*_BET_ and *V*_pore_ are specific surface area and pore volume, respectively. MeOH and TEA stand for methanol and triethanolamine, respectively.

## Supporting Information Available

Characterization of GlyC, sodium 2-methylimidazolate, and reaction byproducts (PDF)
