# Supplementary material for: Synthesis of Zeolitic Imidazolate Framework-8 Using Glycerol Carbonate
Source: ACS Sustain Chem Eng. 2023 Aug 21;11(35):13043–9. doi: 10.1021/acssuschemeng.3c02876 (PMC10481391; doi:10.1021/acssuschemeng.3c02876)
Supplement: Supplementary file 1 — sc3c02876_si_001.pdf [file sc3c02876_si_001.pdf]

Supporting Information

Synthesis of Zeolitic Imidazolate Framework-8 Using Glycerol Carbonate

Masaki Itatani,[a] Norbert Német,[a,b] Nadia Valletti,[c] Gábor Schuszter,[d] Prisco Prete,[e] Pierandrea Lo Nostro,[f] Raffaele Cucciniello,[e,g*] Federico Rossi,[c*] István Lagzi[a,h*]

[a] Department of Physics, Institute of Physics, Budapest University of Technology and Economics, Műegyetem rkp. 3, H-1111 Budapest, Hungary.

[b] Department of Organic Chemistry and Technology, Budapest University of Technology and Economics, Műegyetem rkp. 3, H-1111 Budapest, Hungary.

[c] Department of Physical Sciences, Earth and Environment, Univeristy of Siena, piazzetta Enzo Tiezzi 1, 53100 Siena, Italy.

[d] Department of Physical Chemistry and Materials Science, University of Szeged, Rerrich Béla tér 1, H-6720 Szeged, Hungary.

[e] Department of Chemistry and Biology, University of Salerno, viale Giovanni Paolo II 132, 84084 Fisciano (SA), Italy.

[f] Department of Chemistry "Ugo Schiff", University of Firenze, via della Lastruccia 3, 50019 Sesto Fiorentino (FI), Italy.

[g] Centro Interdisciplinare Linceo Giovani, Accademia Nazionale dei Lincei, Via della Lungara, 10 - 00165 Roma, Italy

[h] ELKH-BME Condensed Matter Research Group, Budapest University of Technology and Economics, Műegyetem rkp. 3, H-1111 Budapest, Hungary.

***Corresponding author**

R.C. rcucciniello@unisa.it; F.R. federico.rossi2@unisi.it; I.L. lagzi.istvan.laszlo@ttk.bme.hu

Number of pages: 11

Number of figures: 9

Number of tables: 1

Table of Contents

• Glycerol carbonate characterization by NMR spectroscopy 3

• Sodium 2-methylimidazolate preparation and characterization 4

• Characterization of the reaction by-products 8

Glycerol carbonate characterization by NMR spectroscopy

The NMR spectra were collected on a Bruker Avance-400 (100MHz ^{13}C) spectrometer using deuterated dimethyl sulfoxide (DMSO) as solvent.

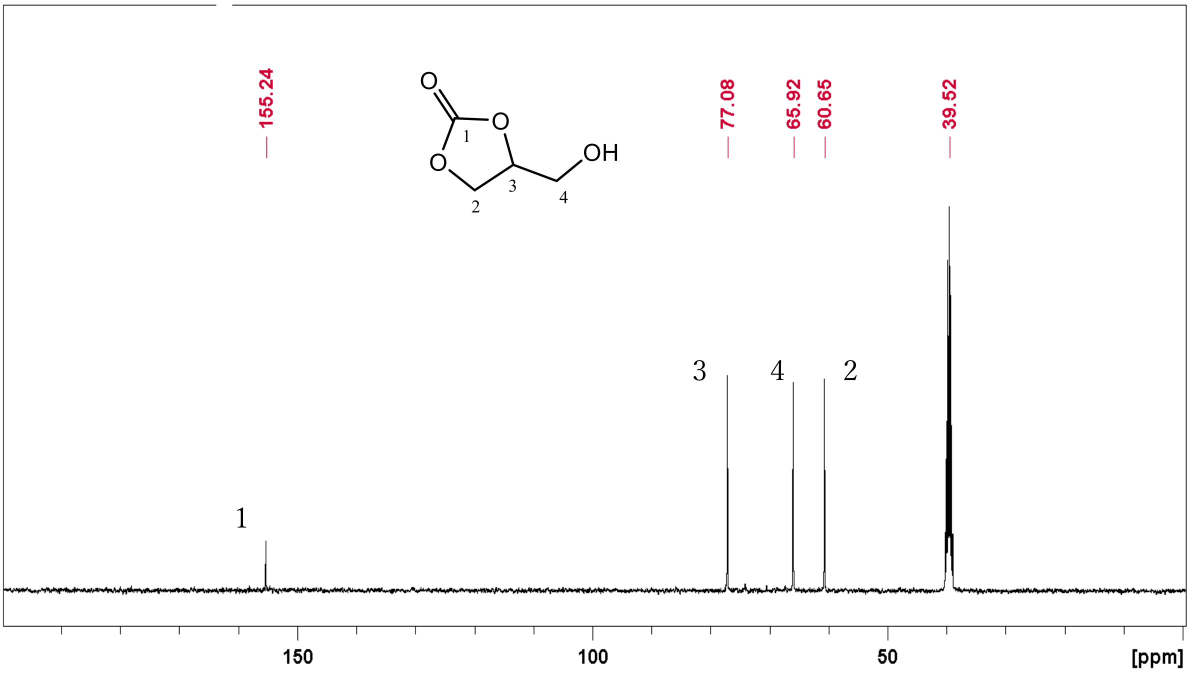

Figure S1. ^{13}C-NMR spectrum of purified glycerol carbonate.

Glycerol carbonate

^{13}C-NMR (100 MHz, DMSO), δ: 155.2 (CO), 77.1 (CH), 65.9 (CH_2), 60.6 (CH_2).

Table S1: Physical properties of Glycerol Carbonate ($C_4H_6O_4$, CAS number = 931-40-8)

**Property**	**Value**
Molecular weight	118.09 g mol^{-1}
Boiling point at 1 bar	627.05 K
Flash point	463.15 K
Vapor pressure at 450.15 K	8 mbar
ρ at 298.15 K	1.4 g cm^{-3}
η at 298.15	0.0854 Pa s

Sodium 2-methylimidazolate preparation and characterization

Sodium imidazolate was prepared through the reaction between 2-methylimidazole and NaOH. In detail, an aqueous solution of 2-methylimidazole (1.0 M) and NaOH (1.1 M) solution was placed under continuous stirring in a closed vial. Reactions were carried out at 25 °C and 100 °C for 24 h and the UV-vis spectrum was recorded with a Varian Cary R 50 UV–vis spectrophotometer after a 1:100 dilution with distilled water. A characteristic signal at 300 nm was recorded and can be attributed to the formation of sodium 2-methylimidazolate. Aqueous solutions of sodium 2-methylimidazolate are characterized by a yellowish color in comparison to a colorless aqueous solution of sole 2-methylimidazole (Figure S2).

UV-vis spectra were also acquired to characterize the solution, after ZIF-8 precipitation, in the optimized reaction conditions ([Hmim] =20 mM, $[Zn(OAc)_2]$ = 10 mM, [NaOH] = 0.01M, 24 h, GlyC as a solvent). ZIF-8 was removed, and a proper amount of the solution was diluted 1:100 with distilled water for UV-vis measurements. As shown in Figure S2, also in this case a strong absorption appeared between 300 nm and 500 nm. A control experiment was carried out using an aqueous solution of 2-methylimidazole. In addition, experiments were also performed using different amounts of base at a fixed concentration of 2-methylimidazole (1.0 M) in water.

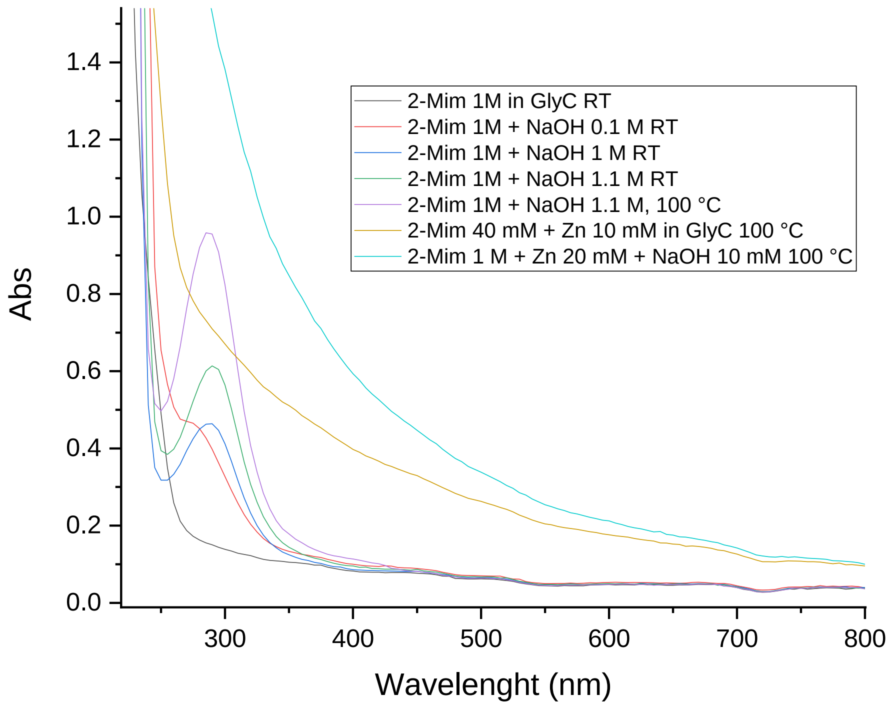

Figure S2. Spectral change of aqueous 2-methylimidazole solutions ([Hmim] = 1000 mM) upon increasing sodium hydroxide concentration and UV-vis spectra of the solution after ZIF-8 precipitation.

Figure S3 illustrates a possible reaction mechanism for the formation of sodium 2-methylimidazolate.

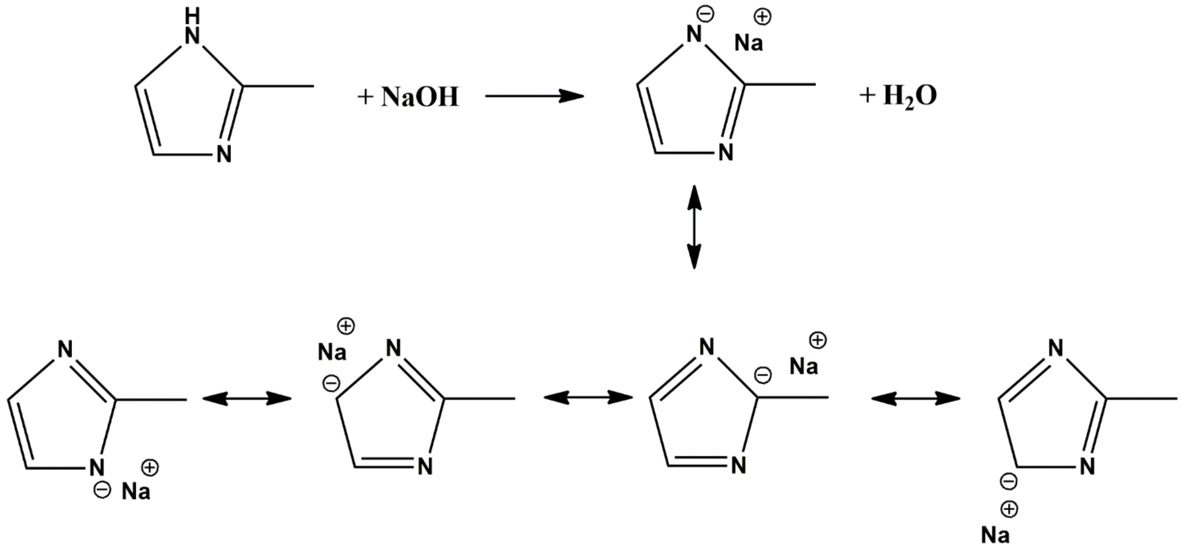

Figure S3. Formation of sodium 2-methylimidazolate from 2-methylimidazole and NaOH.

The formation of sodium 2-methylimidazolate is also confirmed by NMR spectroscopy. The NMR spectrum was collected on a Bruker Avance-400 (300MHz ^1H) spectrometer using CD_3Cl as a solvent and are reported in Figure S4 (red line). ^1H NMR spectra confirm the formation of sodium 2-methyl imidazolate from 2-methylimidazole and NaOH in $CDCl_3$. $CDCl_3$ allows the study of this reaction compared to D_2O where hydrogen-deuterium exchange is predominant. The solution turns from colorless to yellowish color as a consequence of the formation of 2-methylimidazolate. The ^1H NMR spectrum of 2-methylimidazole in CD_3Cl is reported for comparison (blue line).

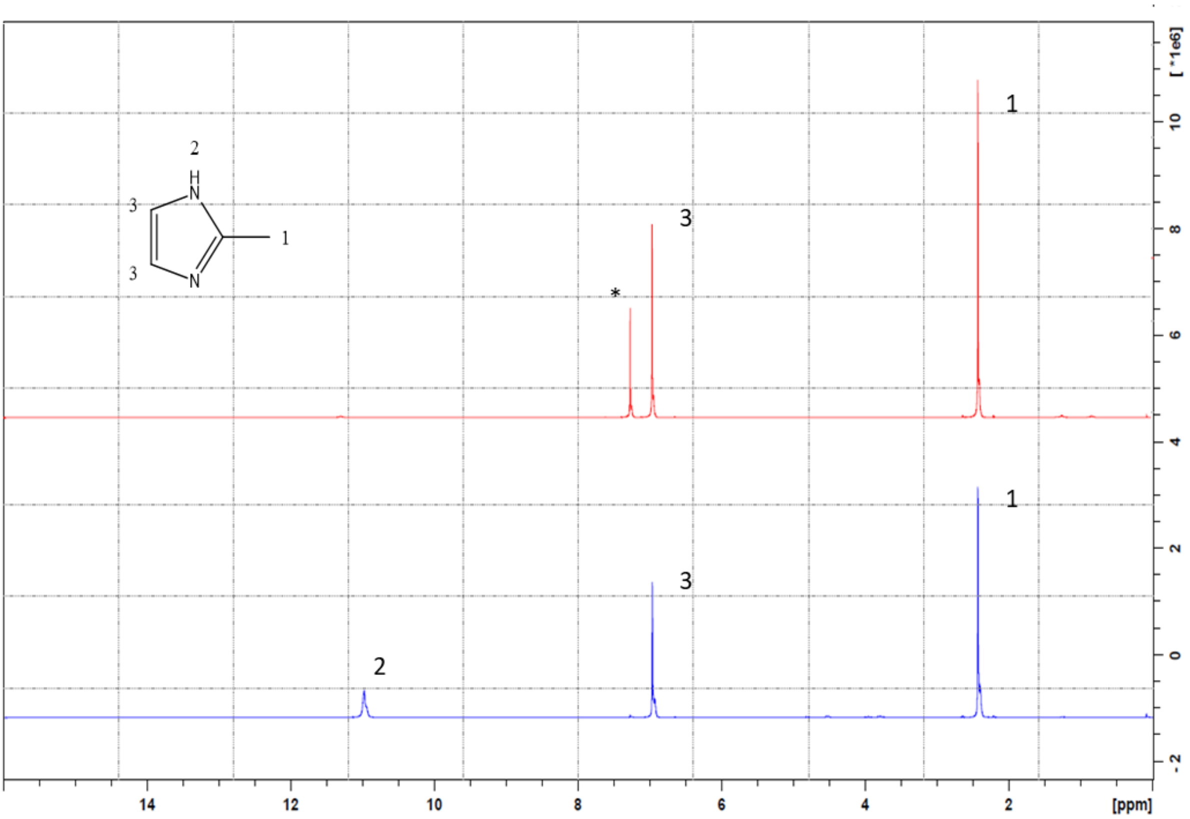

Figure S4. ^1H-NMR spectra of 2-methylimidazole (blue line) and sodium 2-methylimidazolate (red line).

^1H-NMR (300 MHz, $CDCl_3$), δ: 11.2 (2), 7.3 (*) ($CDCl_3$), 6.9 (3), 2.4 (1).

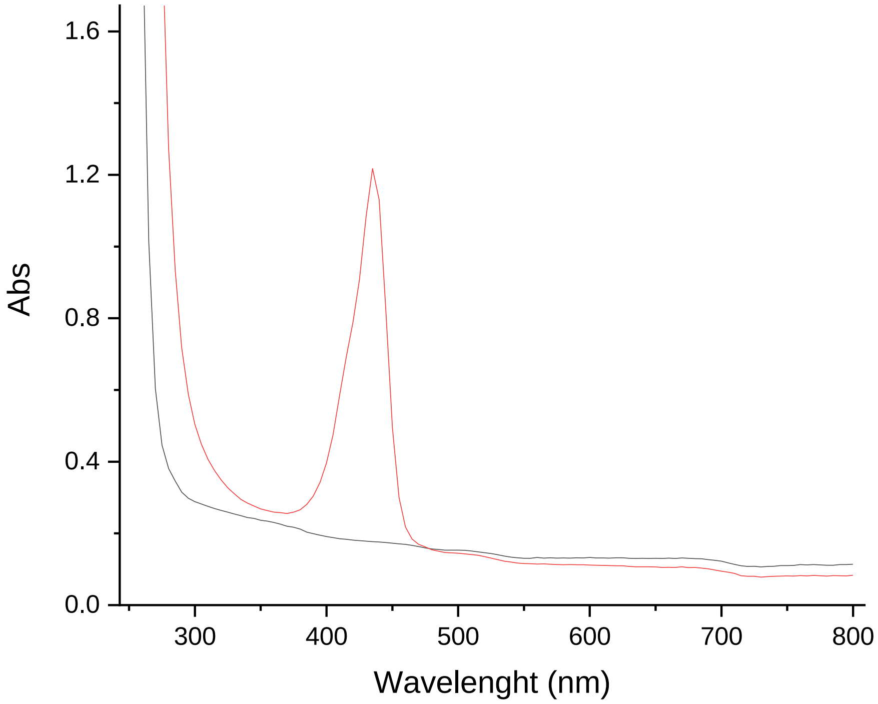

Figure S5. UV-vis spectra of 2-methylimidazole (black line) and 2-methylimidazolate (red line) in chloroform ([Hmim] = 200 mM).

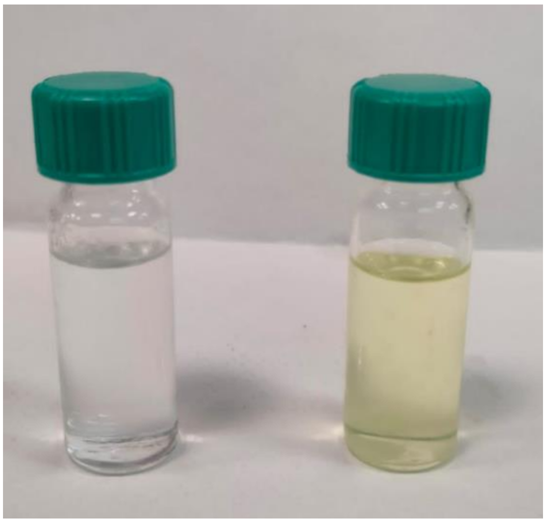

Figure S6. 2-methylimidazole (left side) and 2-methylimidazolate (right side) in chloroform ([Hmim] = 200 mM).

Characterization of the reaction by-products

This section shows and discusses the ^{13}C NMR spectra to gain more insights into the reaction mechanism. The NMR spectra were collected on a Bruker Avance-300 spectrometer. Several experiments were carried out in order to investigate the main side reactions that can take place in the reaction system. First, the reaction of glycerol carbonate with NaOH was investigated. In detail, in a closed glass vial, 5 mL of GlyC and NaOH (0.01 M in GlyC) were mixed at 100 °C for 24 h under magnetic stirring. This reaction yielded glycerol (colorless solution) and carbon dioxide as products (Figure S7), following the reaction mechanism proposed in Figure S8.

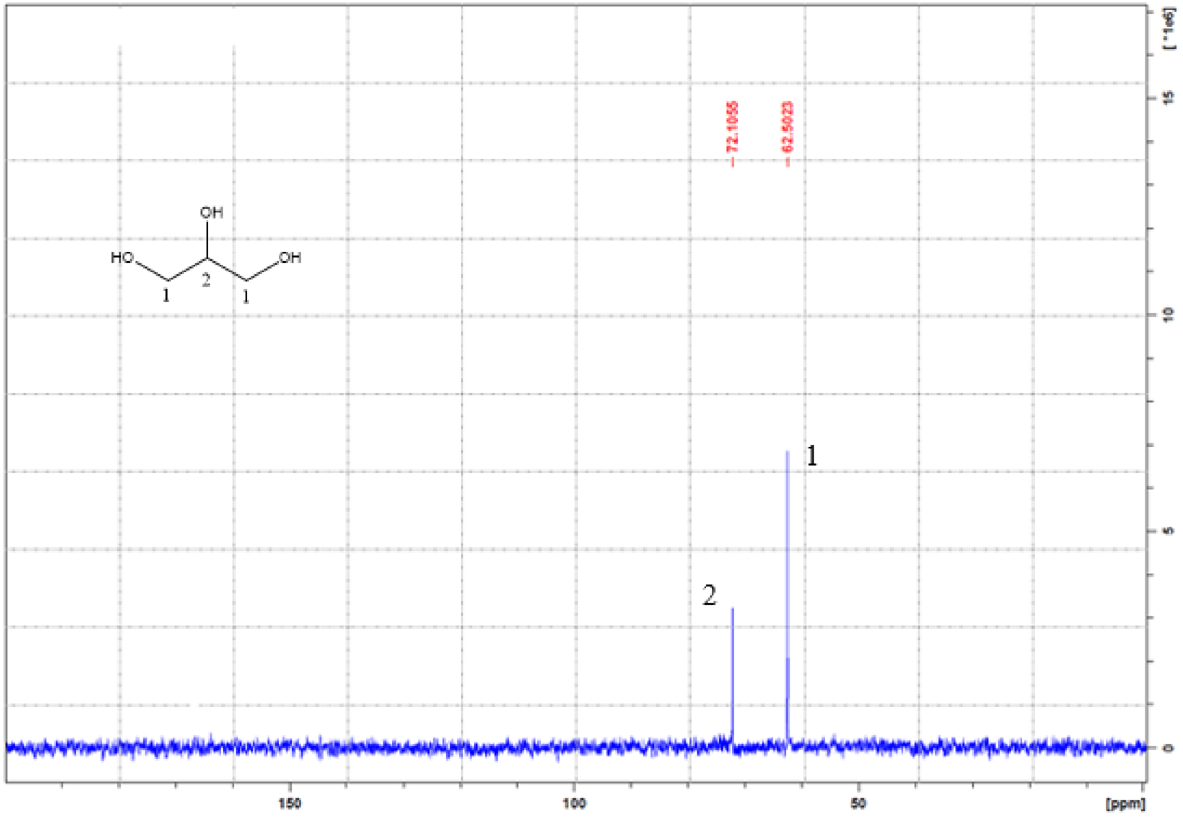

Figure S7. ^{13}C-NMR spectrum of the reaction product (glycerol) obtained by the reaction of glycerol carbonate with NaOH.

^{13}C-NMR (100 MHz, D_2O), δ: 72.1 (CH) (2), 62.5 (CH_2) (1).

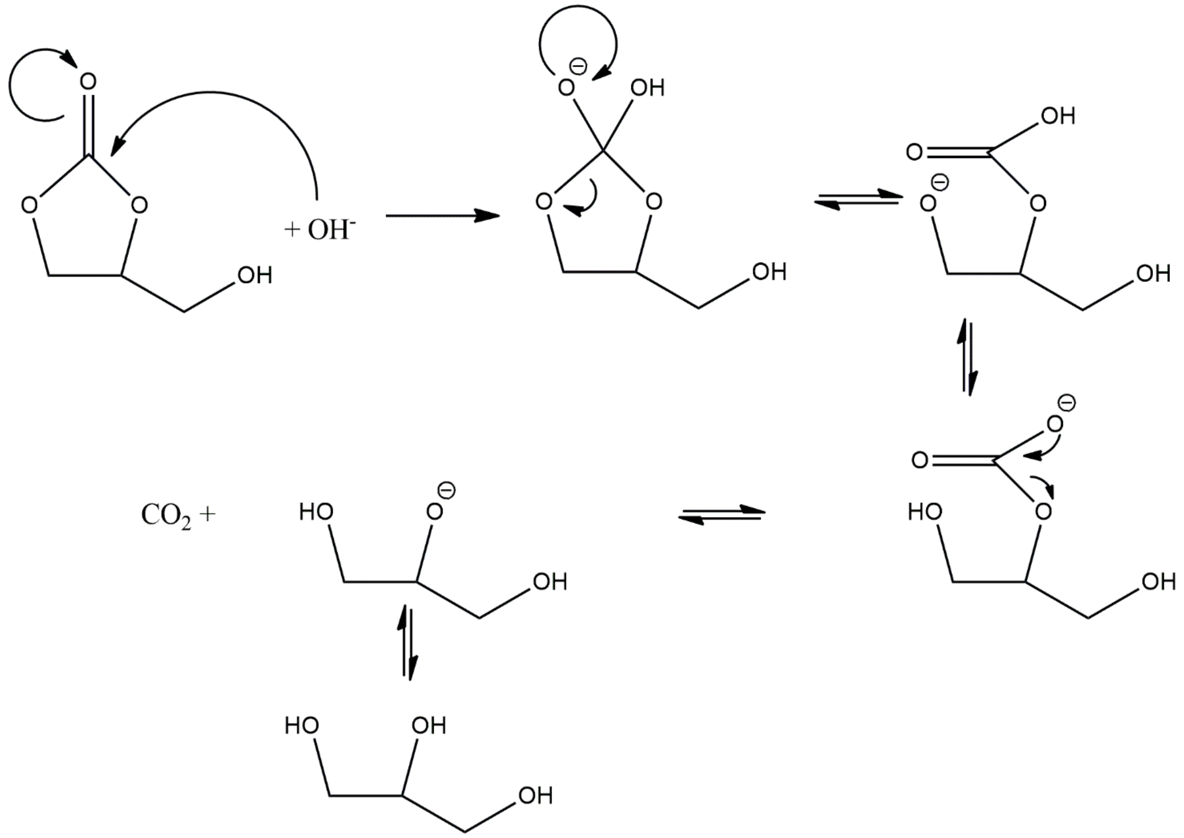

Figure S8. Proposed reaction mechanism for the reaction between glycerol carbonate and NaOH.

For the investigation of the reaction between GlyC and 2-methylimidazole a stoichiometric mixture of GlyC (5 mL) and 2-methylimidazole (1.0 M) was mixed under continuous stirring at 100°C for 24 h in a closed vial. The ^{13}C-NMR spectrum of the reaction product is reported in Figure S10 and compared to that of the solution of 2-methylimidazole (1.0 M) in freshly prepared GlyC (Figure S9). The reaction mixture turned yellowish at 100 °C. Figure S10 reports all signals for both GlyC and 2-methylimidazole. Further signals were identified and will be investigated more in detail in a future work.

The spectrum of the solution after ZIF-8 preparation is not reported due to the excess GlyC (in this case only the characteristic signals of GlyC can be appreciated by NMR spectroscopy). The latter is also related to the low amount of side products formed during the reaction.

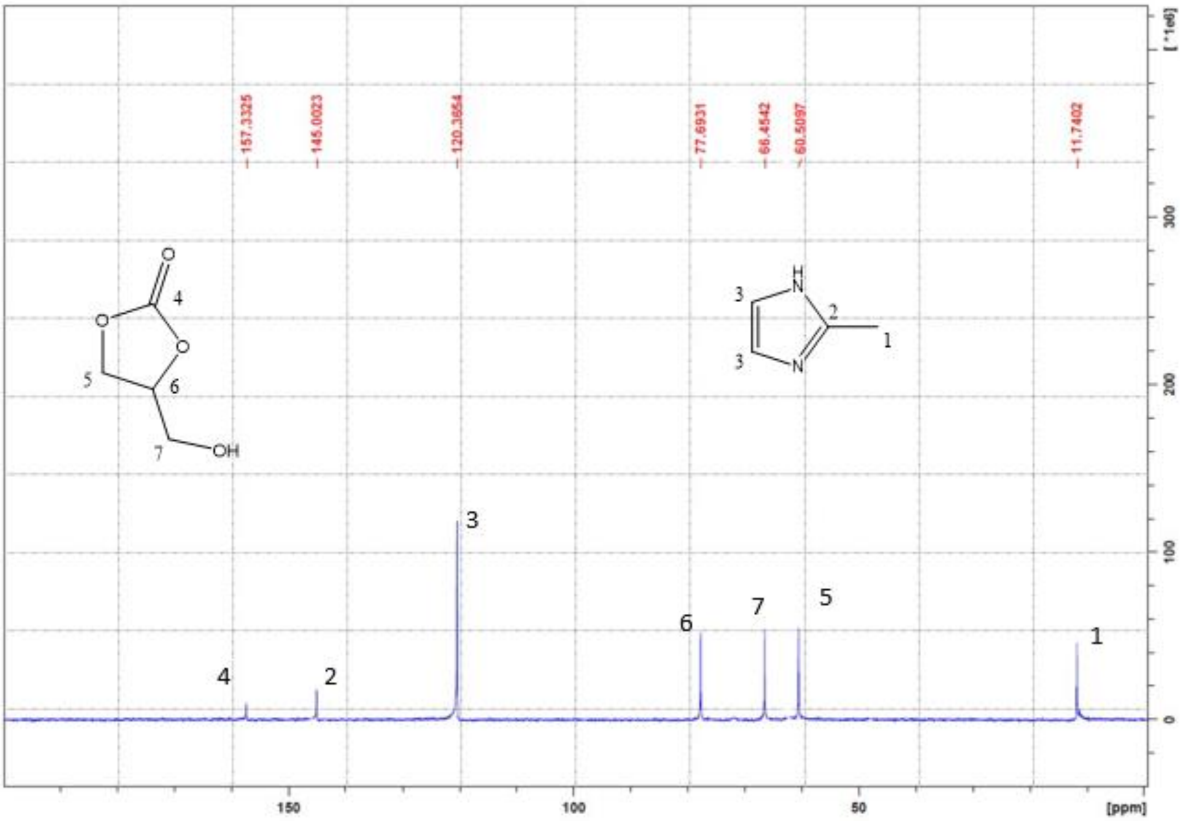

Figure S8. ^{13}C-NMR spectrum of freshly prepared glycerol carbonate and 2-methylimidazole.

^{13}C-NMR (100 MHz, D_2O), δ: 157.3 (CO) (4), 145.0 (CH) (2), 120.3 (CH) (3), 77.7 (CH) (6), 66.4 (CH_2) (7), 60.5 (CH_2) (5), 11.7 (CH_3) (1).

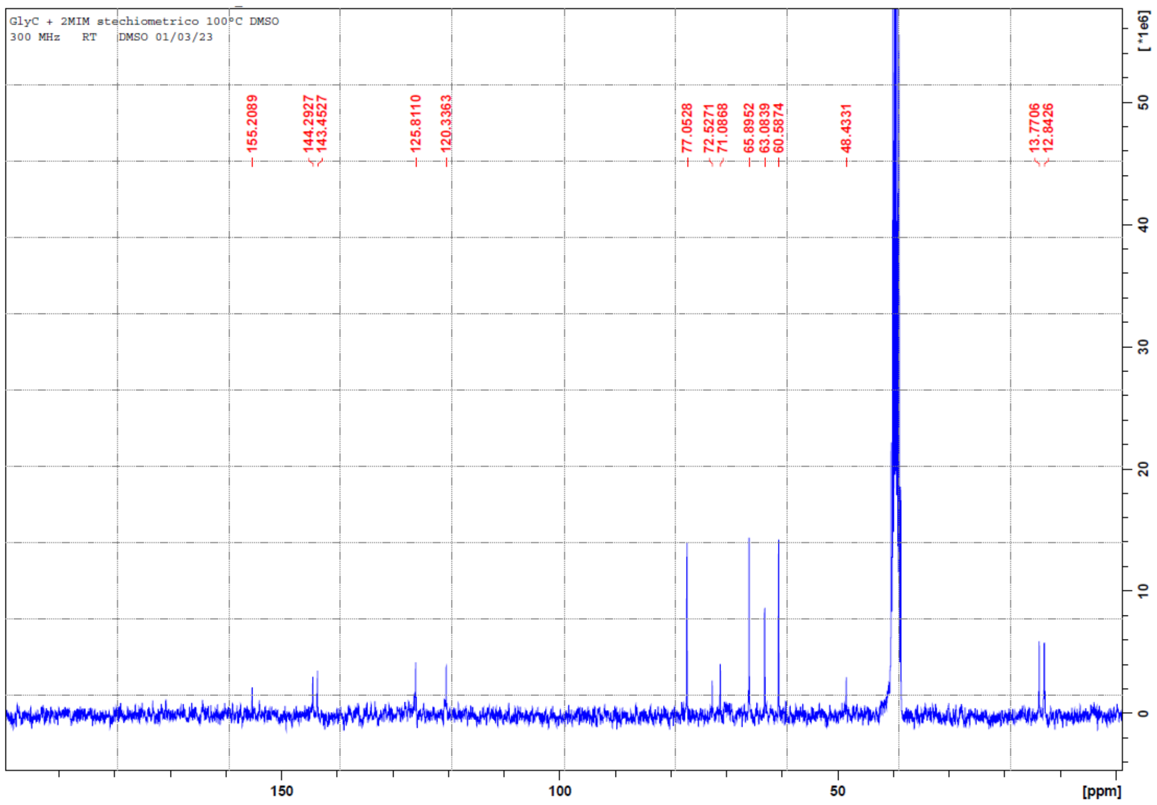

Figure S9. ^{13}C-NMR spectrum to characterize the reaction mixture after the reaction of glycerol carbonate and 2-methylimidazole at 100 °C for 24 h.

^{13}C-NMR (300 MHz, D_2O), δ: 157.3 (CO) (4), 145.0 (CH) (2), 120.3 (CH) (3), 77.7 (CH) (6), 66.4 (CH_2) (7), 60.5 (CH_2) (5), 11.7 (CH_3) (1).
